# Diffusion along perivascular spaces as marker for impairment of glymphatic system in Parkinson’s disease

**DOI:** 10.1038/s41531-022-00437-1

**Published:** 2022-12-21

**Authors:** Ting Shen, Yumei Yue, Fang Ba, Tinging He, Xiaocui Tang, Xingyue Hu, Jiali Pu, Cong Huang, Wen Lv, Baorong Zhang, Hsin-Yi Lai

**Affiliations:** 1 Department of Neurology of the Second Affiliated Hospital, Interdisciplinary Institute of Neuroscience and Technology, Key Laboratory of Medical Neurobiology of Zhejiang Province, Zhejiang University School of Medicine, Hangzhou, China; 2 Department of Neurology of Sir Run Run Shaw Hospital, Zhejiang University School of Medicine, Zhejiang University, Hangzhou, China; 3 Division of Neurology, Department of Medicine, University of Alberta, Edmonton, AB Canada; 4 College of Biomedical Engineering and Instrument Science, Key Laboratory for Biomedical Engineering of Ministry of Education, Zhejiang University, Hangzhou, China; 5 Department of Epidemiology & Health Statistics, School of Public Health, School of Medicine, Zhejiang University, Hangzhou, China; 6 Department of Neurology of the Second Affiliated Hospital, Zhejiang University School of Medicine, Zhejiang University, Hangzhou, China; 7 Department of Sports and Exercise Science, School of Education, Zhejiang University, Hangzhou, China; 8 Department of Medicine and Science in Sports and Exercise, Tohoku University Graduate School of Medicine, Sendai, Japan; 9 MOE Frontier Science Center for Brain Science and Brain-Machine Integration, School of Brain Science and Brain Medicine, Zhejiang University, Hangzhou, China

**Keywords:** Predictive markers, Neurological disorders

## Abstract

The brain glymphatic system is involved in the clearance of misfolding α-synuclein, the impaired glymphatic system may contribute to the progression of Parkinson’s disease (PD). We aimed to analyze the diffusion tensor image along the perivascular space (DTI-ALPS) and perivascular space (PVS) burden to reveal the relationship between the glymphatic system and PD. A cross-sectional study using a 7 T MRI of 76 PD patients and 48 controls was performed to evaluate the brain’s glymphatic system. The DTI-ALPS and PVS burden in basal ganglia were calculated. Correlation analyses were conducted between DTI-ALPS, PVS burden and clinical features. We detected lower DTI-ALPS in the PD subgroup relative to controls, and the differences were more pronounced in patients with Hoehn & Yahr stage greater than two. The decreased DTI-ALPS was only evident in the left hemisphere in patients in the early stage but involved both hemispheres in more advanced PD patients. Decreased DTI-ALPS were also correlated with longer disease duration, higher Unified Parkinson’s Disease Rating Scale motor score (UPDRS III) and UPDRS total scores, as well as higher levodopa equivalent daily dose. Moreover, the decreased DTI-ALPS correlated with increased PVS burden, and both indexes correlated with PD disease severity. This study demonstrated decreased DTI-ALPS in PD, which might initiate from the left hemisphere and progressively involve right hemisphere with the disease progression. Decreased DTI-ALPS index correlated with increased PVS burden, indicating that both metrics could provide supporting evidence of an impaired glymphatic system. MRI evaluation of the PVS burden and diffusion along PVS are potential imaging biomarkers for PD for disease progression.

## Introduction

Parkinson’s disease (PD) is a common neurodegenerative disease that manifests with both motor and non-motor symptoms^1^. The pathological hallmarks of PD are a loss of dopaminergic neurons in substantia nigra pars compacta, and intracellular inclusions containing aggregates of α-synuclein^2^. The aggregation and abnormal deposits of insoluble α-synuclein in the central and peripheral nervous systems^3^ exacerbate the process of neurodegeneration^4^. Impaired α-synuclein clearance system has also been reported in PD, and could be considered as part of the PD pathology^5^.

The glymphatic system is a waste drainage system in the brain, involving the fluid interchange between the interstitial space and perivascular space (PVS)^6^. cerebrospinal fluid (CSF) enters from the para-arterial space into the brain parenchyma. Convective interstitial fluid (ISF) bulk flow is formed to propel waste products towards perivenous space. Subsequently, the waste product is drained out of the brain through the cervical lymphatic system^7,8^. Recent evidence emphasizes the role of the glymphatic system in eliminating neurotoxic protein aggregations in neurodegenerative diseases, such as amyloid-β in Alzheimer’s disease (AD) and α-synuclein in PD^7,9–15^. Glymphatic clearance dysfunction may lead to damage to the dopaminergic neurons secondary to increased α-synuclein deposition. Previous studies have provided more evidence that the blockage of the glymphatic pathway, which leads to drainage dysfunction, could aggravate α-synuclein pathology and exacerbate motor and memory deficits^16^. The glymphatic clearance dysfunction has been considered a possible contributing factor to abnormal α-synuclein aggregation in PD^15^. Conversely, aggregations of misfolding proteins may accumulate around blood vessels and block the glymphatic pathway^17,18^. Hence, PD could be considered a form of “central nervous system interstitial fluidopathy”, a concept used to describe diseases or conditions in which abnormal interstitial fluid dynamics are an important factor^19^. Therefore, evaluating the overall function of the glymphatic system could shed light on the pathophysiological mechanisms of PD^20^.

The glymphatic system has been studied using ex vivo fluorescent microscopic and in vivo two-photon imaging in animal models^7,21^, as well as with intrathecal contrast medium-enhanced MRI^22^ and dynamic PET^23^ in humans. In human studies, the invasiveness and other related risks limited the application of these methods. An improved and non-invasive method to reliably evaluate the function of the glymphatic system is essential.

Recent advances in imaging techniques, including diffusion tensor imaging (DTI), make it possible to assess gross and microstructural changes in vivo. Such assessment in relation to clinical parameters can potentially serve as biomarkers to monitor disease progression. Since the PVS system is an important part of the glymphatic system, previous studies evaluated the glymphatic system indirectly by assessing PVS burden^24,25^. PVS burden in basal ganglia (BG) has been shown to correlate with the severity of PD motor symptoms^25–27^. PVS is a microscopic tubular structure that contains freely moving water molecules^28^. DTI-derived features might be influenced by structural alterations in the glymphatic system. Recently, a non-invasive method, DTI analysis along the perivascular space (DTI-ALPS), has been developed to evaluate the function of brain’s glymphatic system^6^. Water diffusivity along the *x, y*, and *z* axes of the periventricular white matter is generated based on diffusion sequences to calculate the DTI-ALPS index. The DTI-ALPS index represents the water diffusivity along the *x*-axis, which is parallel to the direction of PVS and could partially reflect the activity of the glymphatic system^6,29^. This method has been applied in a series of neurological diseases, including AD^6,30^, PD^29,31^, isolated rapid eye movement sleep behavior disorder^32^, epilepsy^33^, idiopathic normal pressure hydrocephalus^34^, etc. A lower DTI-ALPS index was correlated with increased severity of cognitive impairment in both AD^6,30^ and PD^29^. PD patients showed lower DTI-ALPS index when compared to normal controls^29^, as well as to people with other neurological conditions such as essential tremor^31^. These findings suggest the potential of the DTI-ALPS index as a non-invasive imaging biomarker in evaluating the integrity of the glymphatic system. Ultra-high field 7 T MRI, with increased spatial resolution, contrast and signal-to-noise ratio, improved the visualization and detection rate of PVS, especially small-sized PVS that might be neglected using conventional MRI technology^25,26,35^. Moreover, for DTI studies, 7 T MRI enables visualization of the white matter orientations and making sharp turns into the cortex, which are more difficult to see with the 3 T scanner^36^. The improved higher resolution and reduced partial volume effects make 7 T MRI superior in evaluating DTI-ALPS when compared with other investigations using clinical MRI scanners (1.5 T or 3 T).

The current study aimed to investigate the integrity of the glymphatic system in different stages of PD using potential indicators, including the DTI-ALPS and PVS number/volume indexes derived from ultra-high field 7 T MRI. We evaluated these indexes to test the hypothesis that the diffusivity of perivascular fluid was reduced, and the perivascular structural impairment was increased in PD, which might represent glymphatic dysfunction and structural abnormality, respectively. We also hypothesized that both motor and non-motor symptoms might correlate with the progression of glymphatic impairment. Thus, we planned to further explore the correlation patterns of DTI-ALPS and PVS burden indexes with clinical profiles. Specifically, our study will focus on the role of these biomarkers that reflect the integrity of the glymphatic system, and explore whether impairment of the glymphatic system is correlated with the pathophysiological mechanism of PD.

## Results

### Participant characteristics

Detailed demographic and clinical characteristics of the PD and healthy control (HC) participants are shown in Table 1. PD patients were divided into two subgroups, including the early-stage group (PDa, Hoehn and Yahr (H&Y) stage was lower than or equal to 2) and the more advanced-stage group (PDb, H&Y stage higher than 2). The HCa and HCb were groups of age- and sex-matched controls for PDa and PDb respectively. There were no significant differences between the PDa and HCa groups in education, Hamilton Anxiety Rating Scale (HAM-A) and Mini-Mental State Examination (MMSE) scores. However, individuals in the PDa group showed significantly higher Hamilton Depression Rating Scale (HAM-D) scores than HCa (*p* = 0.03). The PDb group also had significantly higher HAM-D and HAM-A scores than those in the HCb group (*p* < 0.0001 for both scales). Furthermore, there was no significant difference in the Fazekas scores between the PD and HC groups.

**Table 1:** Demographic and clinical characteristics of the participants.

	PD whole (*n* = 76)	HC whole (*n* = 48)	p value^w^	PDa (*n* = 40)	HCa (n = 47)	*p* value^a^	PDb (*n* = 36)	HCb (*n* = 31)	*p* value^b^
Age (years)	57.00 ± 9.53	52.65 ± 9.25	0.01*	54.55 ± 8.34	52.22 ± 8.84	0.25	59.72 ± 10.13	57.86 ± 5.67	0.11
Sex, male (%)	48.7%	41.7%		52.50%	40.43%	0.26	44.44%	35.48%	0.46
Education (years)	7.73 ± 4.90	10.48 ± 5.85	0.01**	8.41 ± 5.01	10.57 ± 5.88	0.09	7.00 ± 4.74	9.03 ± 4.96	0.09
Symptom onset side									
Left-side	31.58%	-	-	30.00%	-	-	33.33%	-	-
Right-side	68.42%	-	-	70.00%	-	-	66.67%	-	-
Disease duration (years)	7.00 ± 4.76	-	-	5.22 ± 4.45	-	-	9.78 ± 6.50	-	-
UPDRS I	2.00 ± 2.17	-	-	1.05 ± 2.06	-	-	2.39 ± 2.26	-	-
UPDRS II	11.57 ± 6.39	-	-	8.85 ± 4.72	-	-	14.58 ± 6.69	-	-
UPDRS III	25.72 ± 12.36	-	-	19.05 ± 11.28	-	-	33.14 ± 8.83	-	-
UPDRS IV	3.13 ± 2.98	-	-	2.03 ± 2.01	-	-	4.36 ± 3.41	-	-
UPDRS total	42.45 ± 19.78	-	-	31.55 ± 16.40	-	-	54.56 ± 15.88	-	-
H&Y stage	2.25 ± 0.87	-	-	1.55 ± 0.45	-	-	3.03 ± 0.45	-	-
LEDD (mg)	605.90 ± 275.40	-	-	504.40 ± 245.35	-	-	715.79 ± 266.66	-	-
HAM-D	7.48 ± 5.83	3.83 ± 3.55	0.0001***	5.56 ± 3.97	3.90 ± 3.57	0.03*	9.56 ± 6.81	3.96 ± 3.43	<0.0001****
HAM-A	6.67 ± 5.49	2.95 ± 2.77	<0.0001****	5.08 ± 5.19	3.00 ± 2.79	0.08	8.39 ± 5.35	3.19 ± 2.77	<0.0001****
MMSE	24.73 ± 4.71	26.33 ± 3.23	0.10	26.23 ± 3.18	26.28 ± 3.26	0.89	23.11 ± 5.54	25.71 ± 3.24	0.09
Fazekas score total	1.82 ± 1.22	1.63 ± 1.10	0.51	1.58 ± 0.83	1.63 ± 1.10	1.00	2.09 ± 1.53	1.83 ± 1.20	0.60
PVWM	1.03 ± 0.65	0.95 ± 0.64	0.57	0.92 ± 0.49	0.95 ± 0.64	0.95	1.15 ± 0.80	1.03 ± 0.68	0.57
DWM	0.79 ± 0.74	0.68 ± 0.66	0.50	0.66 ± 0.58	0.68 ± 0.66	1.00	0.94 ± 0.86	0.79 ± 0.68	0.59

Results are expressed as means ± standard deviation for the continuous variables and as frequencies for the categorical variables.

*PD* Parkinson’s disease, *PDa* PD patients whose H&Y stage was lower than or equal to 2, *PDb* PD patients whose H&Y stage was higher than 2, *HC* healthy control, *UPDRS* unified Parkinson’s disease rating scale, *H&Y stage* Hoehn & Yahr stage, *LEDD* levodopa equivalent daily dose, *HAM-D* Hamilton depression rating scale, *HAM-A* Hamilton anxiety rating scale, *MMSE* mini-mental state examination, *PVWM* periventricular white matter, *DWM* deep white matter.

*indicates *p* value < 0.05; ****indicates *p* value < 0.0001.

^a^indicates comparison between PDa and HCa groups.

^b^indicates comparison between PDb and the matched HCb groups.

^w^indicates comparison between whole PD and HC groups.

### Differences in DTI-ALPS indexes between groups

**Fig. 1:**
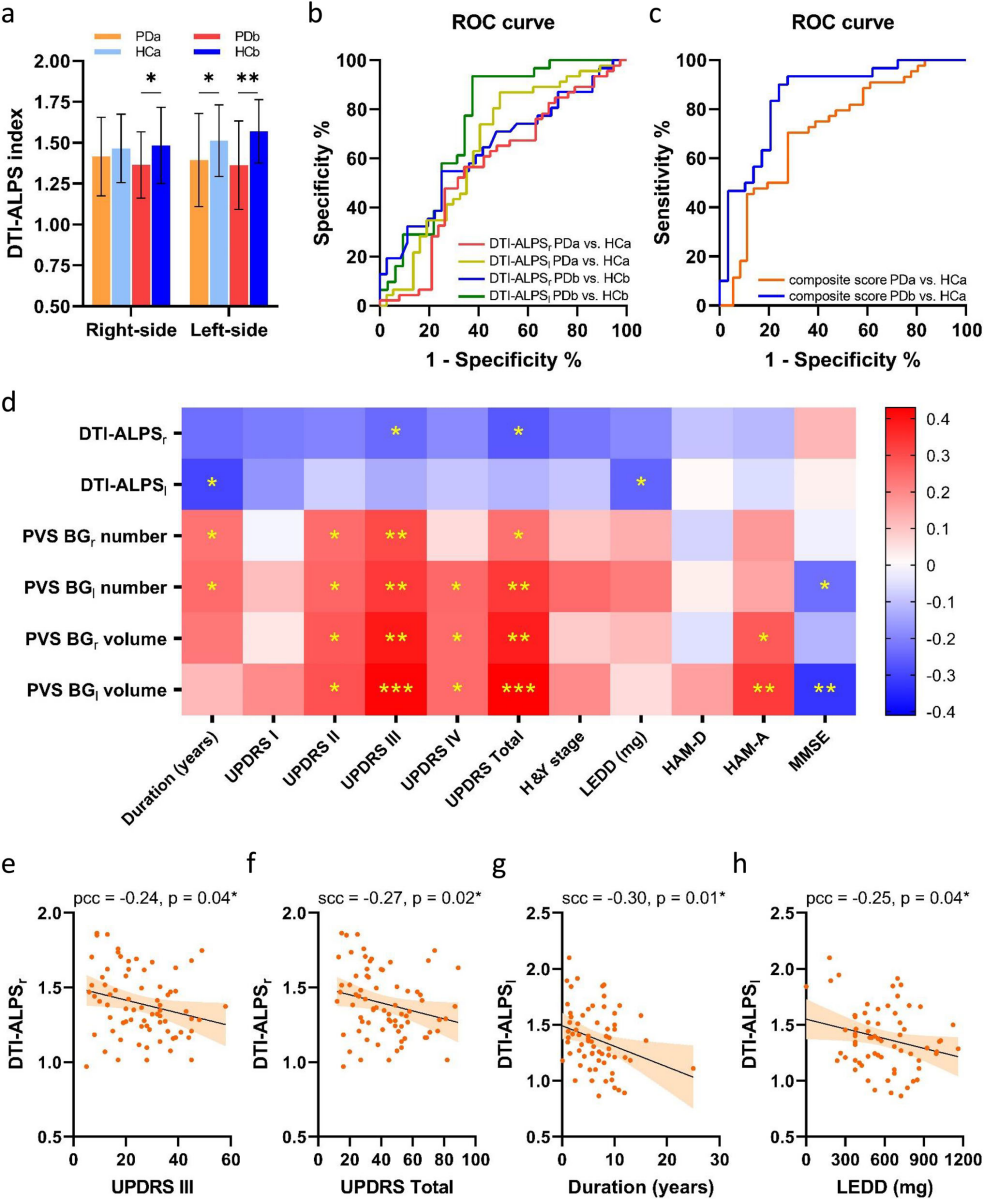
Statistical analysis results of the diffusion tensor image analysis along the perivascular space (DTI-ALPS) indexes. **a** Comparison of DTI-ALPS indexes between PD and HCs. **b** Receiver operating characteristic (ROC) curves for diagnosing PDa and PDb using a single biomarker. **c** ROC curves for diagnosing PDa and PDb using the composite score. **d** Correlations between DTI-ALPS indexes and clinical characteristics in PD. The red and blue colors indicate positive and negative correlations, respectively. **e** Negative correlation between right-hemispheric DTI-ALPS index (DTI-ALPS_r_) and UPDRS III subscore in PD. **f** Negative correlation between DTI-ALPS_r_ and UPDRS total score in PD. **g** Negative correlation between left-hemispheric DTI-ALPS index (DTI-ALPS_l_) and disease duration in PD. **h** Negative correlation between DTI-ALPS_l_ and LEDD in PD. The error bars represent the standard deviation. *indicates *p* value < 0.05, **indicates *p* value < 0.01. PD Parkinson’s disease, PDa PD patients whose H&Y stage was lower than 2, PDb PD patients whose H&Y stage was higher than 2, HC healthy control, UPDRS unified Parkinson’s disease rating scale, H&Y stage Hoehn & Yahr stage, LEDD levodopa equivalent daily dose, HAM-D Hamilton depression rating scale, HAM-A Hamilton anxiety rating scale, MMSE mini-mental state examination, pcc Pearson correlation coefficient, scc Spearman correlation coefficient.

Compared to the HCa group, the PDa group exhibited a significantly lower DTI-ALPS index in the left hemisphere (*p* = 0.04), while no significant difference was found in the right hemisphere (Table 2 and Fig. 1a). The diffusivities along the x-axis in the projection and association fibers, y-axis in the projection fibers and z-axis in the association fibers did not differ between these two groups (Table 2).

**Table 2:** Biomarkers of the glymphatic system in the subgroups of participants.

	PDa (*n* = 40)	HCa (*n* = 47)	*p* value^a^	FDR-adjusted *p* value^a^	PDb (*n* = 36)	HCb (*n* = 31)	*p* value^b^	FDR-adjusted *p* value^b^
DTI-ALPS_r_	1.42 ± 0.24	1.48 ± 0.22	0.32	0.47	1.36 ± 0.20	1.48 ± 0.23	0.03*	0.12
Right Dxassoc (×10^−3^ mm^2^/s)	0.27 ± 0.04	0.27 ± 0.05	1.00	1.00	0.28 ± 0.05	0.27 ± 0.05	0.28	0.44
Right Dxproj (×10^−3^ mm^2^/s)	0.27 ± 0.03	0.29 ± 0.03	0.06	0.17	0.29 ± 0.04	0.29 ± 0.03	0.41	0.57
Right Dzassoc (×10^−3^ mm^2^/s)	0.15 ± 0.03	0.16 ± 0.03	0.74	0.83	0.17 ± 0.04	0.15 ± 0.03	0.12	0.26
Right Dyproj (×10^−3^ mm^2^/s)	0.24 ± 0.05	0.23 ± 0.04	0.20	0.35	0.26 ± 0.04	0.23 ± 0.04	0.01*	0.07
DTI-ALPS_l_	1.39 ± 0.28	1.52 ± 0.22	0.04*	0.14	1.36 ± 0.27	1.57 ± 0.19	0.001***	0.01^#^
Left Dxassoc (×10^−3^ mm^2^/s)	0.26 ± 0.05	0.28 ± 0.04	0.16	0.30	0.28 ± 0.04	0.28 ± 0.04	1.00	1.00
Left Dxproj (×10^−3^ mm^2^/s)	0.27 ± 0.03	0.28 ± 0.03	0.08	0.19	0.28 ± 0.03	0.28 ± 0.03	0.56	0.68
Left Dzassoc (×10^−3^ mm^2^/s)	0.15 ± 0.04	0.15 ± 0.04	0.48	0.64	0.16 ± 0.03	0.14 ± 0.03	0.01*	0.06
Left Dyproj (×10^−3^ mm^2^/s)	0.24 ± 0.04	0.23 ± 0.03	0.15	0.30	0.26 ± 0.05	0.22 ± 0.03	0.0006***	0.02^#^
PVS BG_r_ number	13.15 ± 5.01	11.17 ± 4.28	0.06	0.15	16.30 ± 8.73	11.58 ± 4.50	0.01*	0.05^#^
PVS BG_l_ number	12.06 ± 4.25	12.29 ± 4.67	0.96	1.00	14.91 ± 9.32	13.42 ± 5.04	0.66	0.77
PVS BG_r_ volume	49.83 ± 26.40	42.21 ± 24.51	0.27	0.44	69.60 ± 43.46	39.41 ± 20.33	0.001**	0.01^#^
PVS BG_l_ volume	44.86 ± 26.70	48.93 ± 28.09	0.49	0.62	64.49 ± 43.98	50.65 ± 31.76	0.05	0.16

Results are expressed as means ± standard deviation for the continuous variables.

*PD* Parkinson’s disease, *HC* healthy control, *PDa* PD patients whose H&Y stage was lower than or equal to 2, *PDb* PD patients whose H&Y stage was higher than 2, *DTI-ALPS*_*r*_ right-hemispheric diffusion tensor image analysis along the perivascular space, *DTI-ALPS*_*l*_ left-hemispheric DTI-ALPS, *Dxassoc* diffusivities along the x-axis of ROIs within association fibers, *Dxproj* diffusivities along the x-axis of ROIs within projection fibers, *Dzassoc* diffusivities along the z-axis of ROIs within association fibers, *Dyproj* diffusivities along the y-axis of ROIs within projection fibers, *PVS* perivascular space, *BG*_*r*_ right-hemispheric basal ganglia, *BG*_*l*_ left-hemispheric BG.

*indicates *p* value < 0.05; **indicates *p* value < 0.01; ***indicates *p* value < 0.001.

^#^indicates FDR-adjusted *p* value < 0.05.

^a^indicates comparison between PDa and HCa groups.

^b^indicates comparison between PDb and the matched HCb groups.

When comparing the PDb group with HCb, significantly lower DTI-ALPS indexes were evident in both right and left hemispheres (*p* = 0.03, and *p* = 0.001, respectively) (Table 2 and Fig. 1a). The diffusivities along the *y*-axis in the left-hemispheric projection fibers and *z*-axis in the association fibers, as well as the *y*-axis in the right-hemispheric projection fibers were significantly higher in the PDb group when compared to those of the HCb group (Table 2).

Based on receiver operating characteristics (ROC), the composite score combining the left/right DTI-ALPS and left/right PVS number/volume indexes yielded the best discriminatory performance in distinguishing PDb patients from their age- and sex-matched HCs with the area under ROC curve (AUC) of 0.85, while the AUC of the composite score for distinguishing PDa from HCs was 0.72 (Fig. 1c). When using DTI-ALPS alone, the AUC of left- or right-hemispheric DTI-ALPS alone for distinguishing PDb patients from HCs was 0.75 or 0.64, respectively (Fig. 1b). The AUC of left- and right-hemispheric DTI-ALPS alone to distinguish PDa patients from HCs was 0.65 and 0.57 (Fig. 1b), respectively. Therefore, the yield of differentiating PD from HCs increased by using the composite score compared to using a single biomarker.

### Correlations of DTI-ALPS indexes with clinical characteristics

Correlation analysis between the MRI indexes and clinical characteristics was performed in all PD patients (Table 3). In brief, DTI-ALPS indexes were negatively correlated with disease duration, Unified Parkinson’s Disease Rating Scale (UPDRS) I, II, III, IV subscores, UPDRS total score, Hoehn & Yahr stage (H&Y stage), levodopa equivalent daily dose (LEDD)^37^, HAM-A, and HAM-D scores (Fig. 1d). Additionally, DTI-ALPS indexes showed a positive trend with MMSE score (Fig. 1d). The DTI-ALPS index in the right hemisphere was negatively correlated with UPDRS III (pcc = −0.24, *p* = 0.04, Fig. 1e) and UPDRS total score (scc = −0.27, *p* = 0.02, Fig. 1f). In the left hemisphere, DTI-ALPS index negatively correlated with disease duration (scc = −0.30, *p* = 0.01, Fig. 1g) and LEDD (pcc = −0.25, *p* = 0.04, Fig. 1h). Correlations between PVS burden and clinical profiles are shown in Table 3. PVS number/volume in BG was also found to be positively correlated with UPDRS scores. PVS numbers in bilateral BG were positively correlated with disease duration. PVS volume in bilateral BG was positively correlated with the HAM-A score. PVS number and volume in the left-hemispheric BG both negatively correlated with the MMSE score.

**Table 3:** Correlations between clinical features and biomarkers of the glymphatic system in all PD patients.

	Duration	UPDRS I	UPDRS II	UPDRS III	UPDRS IV	UPDRS Total	H& Y stage	LEDD	HAM-D	HAM-A	MMSE	Fazekas score total	Fazekas score PVWM	Fazekas score DWM	DTI-ALPS_r_	DTI-ALPS_l_
DTI-ALPS_r_	−0.23 (0.05)	−0.21 (0.07)	−0.20 (0.09)	−0.24^a^ (0.04*)	−0.19 (0.11)	−0.27 (0.02*)	−0.22 (0.09)	−0.19^a^ (0.11)	−0.10 (0.41)	−0.12 (0.33)	0.12 (0.31)	−0.22 (0.07)	−0.18 (0.14)	−0.25 (0.04*)	-	-
DTI-ALPS_l_	−0.30 (0.01*)	−0.17 (0.16)	−0.08 0.53)	−0.14^a^ (0.27)	−0.09 (0.44)	−0.12 (0.33)	−0.09 (0.34)	−0.25^a^ (0.04*)	0.01 (0.94)	−0.06 (0.66)	0.02 (0.85)	−0.08 (0.53)	−0.11 (0.40)	−0.04 (0.73)	0.36 (0.002**)^#^	-
PVS BG_r_ number	0.24 (0.05*)	−0.02 (0.88)	0.25 (0.04*)	0.30^a^ (0.009**)	0.06 (0.61)	0.24 (0.04*)	0.10 (0.53)	0.14^a^ (0.26)	−0.07 (0.56)	0.17 (0.15)	−0.02 (0.84)	0.17 (0.13)	0.18 (0.14)	0.18 (0.16)	−0.37^a^ (0.002**)^#^	−0.10 (0.43)
PVS BG_l_ number	0.25 (0.04*)	0.11 (0.37)	0.26 (0.02*)	0.33 (0.004**)^#^	0.25 (0.03*)	0.33 (0.004**)^#^	0.25 (0.11)	0.22 (0.06)	0.03 (0.81)	0.15 (0.21)	−0.23 (0.05*)	0.20 (0.04*)	0.25 (0.05)	0.23 (0.09)	−0.47 (<0.0001****)^#^	−0.02 (0.88)
PVS BG_r_ volume	0.23 (0.06)	0.04 (0.74)	0.28 (0.02*)	0.39 (0.001**)^#^	0.25 (0.03*)	0.38 (0.001**)^#^	0.09 (0.57)	0.12 (0.33)	−0.05 (0.67)	0.28 (0.02*)	0.12 (0.31)	0.15 (0.13)	0.18 (0.13)	0.18 (0.20)	−0.26 (0.03*)	0.15 (0.25)
PVS BG_l_ volume	0.12 (0.33)	0.19 (0.10)	0.29 (0.01*)	0.42 (0.0002***)^#^	0.25 (0.03*)	0.43 (0.0002***)^#^	0.20 (0.21)	0.06 (0.62)	0.16 (0.18)	0.33 (0.004**)^#^	0.33 (0.005**)^#^	0.16 (0.08)	0.21 (0.07)	0.22 (0.17)	−0.26 (0.03*)	0.10 (0.41)

Results are expressed as correlation coefficients (upper value) and associated *p* values (lower values).

*PD* Parkinson’s disease, *UPDRS* unified Parkinson’s disease rating scale, *H&Y* stage Hoehn & Yahr stage, *LEDD* levodopa equivalent daily dose, *HAM-D* Hamilton depression rating scale, *HAM-A* Hamilton anxiety rating scale, *MMSE* mini-mental state examination, *DTI-ALPS*_*r*_ right-hemispheric diffusion tensor image analysis along the perivascular space, *DTI-ALPS*_*l*_ left-hemispheric DTI-ALPS, *PVS* perivascular space, *BG*_*r*_ right-hemispheric basal ganglia, *BG*_*l*_ left-hemispheric BG, *PVWM* periventricular white matter, *DWM* deep white matter.

*indicates *p* value < 0.05; **indicates *p* value < 0.01; ***indicates *p* value < 0.001; ****indicates *p* value < 0.0001.

^#^indicates FDR-adjusted *p* value < 0.05.

^a^indicates the Pearson correlation coefficient, while others are Spearman correlation coefficients.

### Correlations between neuroimaging biomarkers

**Fig. 2:**
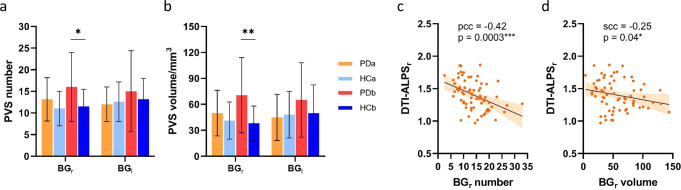
Statistical analysis results of the PVS burden. Comparison of the PVS number (**a**) and volume (**b**) between the PD and HC groups. Correlations of right-hemispheric DTI-ALPS index with PVS number (**c**) and PVS volume (**d**) in right BG. The error bars represent the standard deviation. *indicates *p* value < 0.05, **indicates *p* value < 0.01; ***indicates *p* value < 0.001. PVS perivascular space, PD Parkinson’s disease, HC healthy control, PDa PD patients whose H&Y stage was lower than or equal to 2, PDb PD patients whose H&Y stage was higher than 2, DTI-ALPS_r_ right-hemispheric diffusion tensor image analysis along the perivascular space, BG_r_ right-hemispheric basal ganglia, scc Spearman correlation coefficient.

To investigate whether the alteration of the DTI-ALPS index was consistent with increased PVS burden in PD, the correlations between the two metrics were calculated in all PD patients (Table 3). PVS number (*p* = 0.01) and volume (*p* = 0.001) in the right BG were significantly higher in PDb patients compared to HCb (Fig. 2a, b). Significant negative correlations were found between the DTI-ALPS index of the right hemisphere with the ipsilateral PVS number (pcc = −0.42, *p* = 0.0003, Fig. 2c) and PVS volume (scc = −0.25, *p* = 0.04, Fig. 2d) in BG. Furthermore, DTI-APLS of the right hemisphere was negatively correlated with the DWM Fazekas score (scc = −0.25, *p* = 0.04). Although only the PVS number in the left BG was significantly related to the total Fazekas score (scc = −0.20, *p* = 0.04), PVS burden indexes showed a trend of positive correlation with the Fazekas score.

## Discussion

The DTI-ALPS index is a recently established non-invasive method to evaluate brain glymphatic function. In this study, we utilized higher resolution 7 T MRI, a superior tool in evaluating fiber tracks for DTI-ALPS and detecting small/normal-sized PVSs. We detected lower DTI-ALPS in both PD subgroups compared to age- and sex-matched HCs. We also observed a left/right differentiation of the DTI-ALPS index in PD patients, and a possible progression impacting bilateral hemispheres as the disease progresses. The lower DTI-ALPS indexes were correlated with increased disease severity, as reflected by UPDRS and LEDD. Furthermore, PVS burden was inversely correlated with DTI-ALPS indexes. Therefore, the DTI-ALPS indexes could be mutually complementary with the PVS burden, which could be used to evaluate the function of the glymphatic system.

This study investigated the brain glymphatic system in PD with both DTI-ALPS and PVS burden indexes derived from 7 T MRI, and analyzed bilateral hemispheres separately. The DTI-ALPS index is calculated as the ratio of the diffusion in the perivascular direction along the medullary veins to the diffusion that is perpendicular to the principal fiber track direction^6,31^. Although the DTI-ALPS was established to be measured in the periventricular space and evaluated the diffusion aberrations near PVS, it can represent the overall glymphatic function to some extent^6,31^.

Chen et al. calculated the DTI-ALPS index in the dominant left hemisphere and observed a lower index in PD patients at different disease stages^29^. In our study, only left-hemispheric glymphatic impairment was evident in the early disease course, while both hemispheres showed impairments in the PDb group as the disease advanced (H&Y >2). However, the left-hemispheric DTI-ALPS changes were more pronounced. This may suggest that glymphatic impairment starts from the left brain and propagates to the right through the progression of PD, although it potentially relates to the right hemibody being the location of motor symptom onset in nearly 70% of PD patients in this study. As the disease advances, such changes gradually progress to involve both hemispheres. This might be the reason that no significant differences were observed in DTI-ALPS indexes between right-onset and left-onset PD patients in our study cohort (Supplementary Table 1). Previous studies have provided evidence to support the characteristic asymmetry of motor symptoms, brain network connectivity, and epigenetic patterns in PD, especially in early stage^38–40^. Since all the included subjects in our study were right-handed, the left hemisphere was the dominant brain. Higher DNA methylation levels in cortical neurons of the left hemisphere were observed, and PD patients exhibited more hemispheric asymmetry than controls^40^. The left hemisphere was more vulnerable to aging-related epigenetic alterations in PD, in agreement with previous neuroimaging findings that demonstrated a pattern of early left-, and late right-hemispheric cortical atrophy^38^. Furthermore, the left-hemispheric DTI-ALPS was correlated with disease duration, indicating that the diffusion aberrations near PVS could be an ongoing process in PD as the disease progresses. The negative correlations between the DTI-ALPS index and the UPDRS suggest a link between the diffusion along PVS and disease status. There was a correlation between decreased DTI-ALPS and anxiety, depression, and cognitive defects in our study, suggesting a possible role of these indexes as imaging markers to reflect the progression of neuropsychiatric dysfunction in PD, potentially before motor symptom onset^32^. In patients with more severe cognitive defects, such as AD patients^6^ and PD dementia^29^, significantly lower ALPS index and its inverse correlation with cognitive scores were observed, which is consistent with our findings.

The PVS system is part of the glymphatic system, both structurally and functionally. Through the PVS, harmful metabolites are cleared from the brain^41^. Similarly, glymphatic dysfunction may lead to PVS enlargement and accumulation of misfolded protein aggregates. An increased PVS burden indicates dysfunctional endothelial walls and impaired ISF exchange^41^, and would further lead to glymphatic dysfunction. Our previous studies have shown a higher PVS burden in PD patients^25,35^, with such burden in BG as a candidate biomarker to evaluate PD motor severity^24^. Increased PVS burden is one of the manifestations of an impaired perivascular clearance system, and mainly indicates a structural abnormality in the glymphatic system. As an approximate measure of glymphatic flow, DTI-ALPS could indirectly represent the transport function of the glymphatic system. Higher PVS burden and lower DTI-ALPS indexes both represent impairments in the glymphatic system. We observed significant negative correlations between DTI-ALPS and PVS burden indexes in the current study. Although the predominant alterations of DTI-ALPS and PVS burden indexes were found in different hemispheres, these structural and functional measures might be interrelated and could mutually complement each other in evaluating the glymphatic system in PD. In addition, significant correlations were only found with the PVS number in BG, indicating that the PVS number rather than volume is more representative of the glymphatic function. PVS number in BG was significantly correlated to the PD disease severity and LEDD^24^. The DTI measures of certain subcortical nuclei and white matter fibers within or nearby the BG also significantly correlated with the ipsilateral PVS burden^24^. PVS number has been proven to be a more important measurement to represent the severity of motor symptoms in PD^24^.

The DTI-ALPS index provides an approximate measure of the glymphatic activity. Its alteration might be affected by other factors other than glymphatic dysfunction. Pathological findings of small vessel diseases, including white matter lesions (WMLs), lacunas, and microbleeds were found to be associated with DTI-ALPS index^32,42^. Similarly, brain atrophy, tortuous or enlarged medullary veins, axonal degeneration, and demyelination might also affect the water diffusion in brain tissues, which influences the value of the DTI-ALPS index. WHLs are well-known biomarkers of small vessel disease^43^, commonly found around enlarged PVSs and demonstrated to be related with PVS burden^8,44,45^. WML severity was further assessed in this study using the semi-quantitative Fazekas rating scale^46^. Although there was no significant difference in the Fazekas scores between PD and HC groups in the current study, we also observed an association between the DTI-ALPS index and Fazekas score as recently reported^31^. WML worsened along with the reduced DTI-ALPS and increased PVS burden. Induced dilation of PVS and glymphatic dysfunction in animal models might drive axonal/white matter damage and cognitive decline^47^. WMLs are recognized as pathological characteristics of neurovascular-glymphatic dysfunction and contributors to gait dysfunction and cognitive decline^48^. Similar to PVS, WMLs might be both the cause and consequence of glymphatic dysfunction^48^.

DTI-ALPS index could be regarded as a non-invasive biomarker to measure the fluid dynamics in the brain and represent the potential glymphatic function, as well as offer insight into fluidopathy in PD. To a certain extent, decreased DTI-ALPS indexes are linked to impairment of glymphatic function^6,31^, and its concomitant higher PVS burden suggests possible blockage of the glymphatic pathway. In PD, the failure of perivascular clearance may have led to α-synuclein deposition and the formation of a Lewy body^15,49^. Recent evidence has provided further insight into the role of the glymphatic system and its related pathways in neurodegenerative diseases. Since the waste clearance activity in the brain increases by 60% during the sleep state^8^, sleep disturbances might lead to suppressed glymphatic function. Thus, investigating the relationship between the brain’s glymphatic system and sleep could be meaningful in understanding the interstitial fluidopathy of PD. As the strongest prodromal marker of α-synucleinopathy disease, rapid eye movement sleep behavior disorder exhibited decreased DTI-APLS, indicating the presence of glymphatic system dysfunction^32^. Furthermore, oxidative stress triggers a cascade of events that lead to the degeneration process of dopaminergic neurons in PD^50,51^. Increased oxidative stress might also impair the convective flow and CSF-to-ISF turnover, which causes glymphatic dysfunction^29,52^. DTI-ALPS was shown to be associated with oxidative burden conditions in PD, indicating the possible role of oxidative stress in the glymphatic system^29^. These risk factors and pathogenic pathways possibly contribute to the impairment of the glymphatic system, which are yet to be studied in future studies.

The strength of this study includes using both DTI-ALPS and PVS burden indexes to assess the glymphatic functional and structural abnormality, and to assess the asymmetry of fluidopathy in the brain using ultra-high field 7 T MRI. The 7 T MRI improved the visualization of PVS^25^ and provided better-resolution DTI images to calculate the DTI-ALPS index. We report two candidate imaging biomarkers, DTI-ALPS index and PVS, which were complementary in evaluating the glymphatic function, and thus provides a more complete assessment. There are some limitations to the current study. First, we conducted this study in a single center, and the sample size was relatively small for subgroup analysis, which needs further verification in future multi-center study with a larger sample size. Second, we speculated, based on our results, that the glymphatic dysfunction might initiate from the left hemisphere and progress to the right hemisphere in our study sample, since close to 70% of the PD patients had right-sided symptom onset. However, no differences were observed in DTI-ALPS indexes between right-onset and left-onset PD patients. Whether the left to right propagation of the glymphatic dysfunction stands and the possible presentation of developing synucleinopathy cannot be concluded from our results, and long-term prospective studies are needed. Thus, future research is warranted, which will help to build the multi-modality biomarker profile for PD.

In conclusion, this study demonstrated that the DTI-ALPS indexes were decreased in PD, which might initiate from the left hemisphere and progressively involve the right hemisphere with the disease progression. Decreased DTI-ALPS index correlated with increased PVS burden, indicating that both metrics could reflect brain glymphatic function. MRI evaluation of the diffusion aberrations near PVS potentially could be an imaging biomarker for PD disease progression in evaluating both motor and non-motor features.

## Methods

### Participants and clinical data collection

A total of 76 PD patients were recruited from the Second Affiliated Hospital, and Sir Run Run Shaw Hospital of Zhejiang University School of Medicine. All PD patients were evaluated by movement disorders neurologists. The diagnosis was based on the International Parkinson & Movement Disorder Society (MDS) Clinical Diagnostic Criteria for PD^1^. A total of 48 community volunteers without neurological or psychiatric disorders were recruited as HCs. HCs had no family history of PD or related neurodegenerative disorders. The PD patients were divided into two subgroups according to their Hoehn & Yahr stage (H&Y stage). PDa group included 40 patients whose H&Y stage was lower than or equal to 2, while the PDb group contained 36 patients with H&Y stage higher than 2. To match for age and sex for the PDa and PDb groups, 47 controls were assigned to HCa, and 31 control participants were assigned to HCb group. Some HCs overlapped between the two groups.

For clinical evaluation, PD patients were assessed using UPDRS I to IV and H&Y stage during defined off-stage (PD medications on hold for a minimum of 12 h). We applied HAM-A and HAM-D to assess anxiety and depression. The MMSE was used to evaluate cognitive function during the on-stage of patients.

The study was approved by the ethics committee of the Second Affiliated Hospital of Zhejiang University School of Medicine (Ethics No. 2015-081) and the ethics committee of Sir Run Run Shaw Hospital of Zhejiang University School of Medicine (Ethics No. 20200908-30), respectively. Written informed consent was obtained from all the participants.

### MRI images acquisition

MRI images were acquired with a 7 T Magnatom research system (Siemens Healthcare, Erlangen, Germany) with prototype sequences, including a magnetization prepared with two rapid gradient echoes (MP2RAGE) sequence (voxel size: 0.7 mm^3^ × 0.7 mm^3^ × 0.7 mm^3^, TR = 5000 ms, TI1/TI2 = 900/2750 ms, TE = 2.3 ms, α1/α2 = 5°/3°, and two times generalized autocalibrating partial parallel acquisition acceleration), a T2-weighted turbo spin echo sequence (voxel size: 0.5 mm^3^ × 0.5 mm^3^ × 2.4 mm^3^, TR = 7000 ms and TE = 66 ms), a multiband echo-planar DTI sequence (voxel size: 1.5 mm × 1.5 mm × 1.5 mm, TR = 6000 ms, TE = 71.8 ms, b-values including 0, 1000, and 3000 s/mm^2^), and a susceptibility-weighted imaging (SWI) sequence (voxel size: 0.25 mm × 0.25 mm × 1 mm, TR = 30 ms and TE = 15 ms). Patients underwent MRI scan during defined off-stage (PD medications on hold for a minimum of 12 h).

### DTI-ALPS processing

**Fig. 3:**
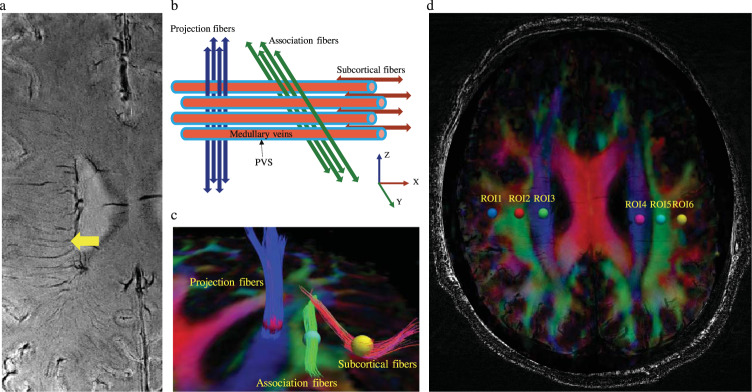
Illustration of the diffusion tensor image analysis along the perivascular space (DTI-ALPS) method. **a** Axial susceptibility-weighted imaging (SWI) map at the level of the lateral ventricle body showing the medullary veins that run perpendicular to the ventricular wall (yellow arrow). **b** Schematic drawing of the spatial relationships between perivascular space and the white matter fibers. **c** Fiber tracking of projection, association, and subcortical fibers. **d** Fusion of SWI and color-coded fractional anisotropy (FA) maps. Six regions of interests (ROIs) are located in the areas of projection fibers, association fibers, and subcortical fibers (sphere).

The DTI data were processed using FSL (version 6.0.1, FMRIB Software Library; http://www.fmrib.ox.ac.uk/fsl) to conduct processing steps, including topup correction, eddy current correction, DTIFIT, registration to standard space. Color-coded fractional anisotropy (FA) maps and diffusivity maps in directions of the x-, y-, and z-axes (Dx, Dy, Dz) were generated. DTI-ALPS method was applied to evaluate the activity of the glymphatic system^6,29^. At the level of the lateral ventricle body, the SWI image showed that the medullary veins ran perpendicular to the ventricular wall (Fig. 3a) and were along with the PVS (x-axis). The projection fibers next to the lateral ventricle ran in the z-axis direction, the association fibers ran in the y-axis direction, and the subcortical fibers ran along with the PVS in the x-axis direction (Fig. 3b). Deterministic tractography was conducted using Diffusion Toolkit version 0.6.4 and TrackVis version 0.6.1 (http://trackvis.org) to visualize the projection, association and subcortical fibers that run perpendicular to each other (Fig. 3c). On the color-coded FA map, we placed spherical region of interests (ROIs) within the projection fibers, association fibers and subcortical fibers respectively in bilateral hemispheres (Fig. 3d), and diffusivities along different directions of these ROIs were extracted. The diffusivities along the x-axis of ROIs within projection (Dxproj) and association (Dxassoc) fibers areas could partially represent water diffusivity along the PVS with minimal influence from the nerve fibers^6^. The diffusivities along the y-axis of projection fibers (Dyproj) and along the z-axis of association fibers (Dzassoc) were also extracted. The major difference for the water molecule behavior between the mean diffusivity along x-axis in projection (Dxproj) and association (Dxassoc) fibers, and mean diffusivity along the y-axis in projection fibers (Dyproj) and along the z-axis of association fibers (Dzassoc) might come from the involvement of PVS^6^. The DTI-ALPS index was defined as follows: ALPS index = mean (Dxproj, Dxassoc)/mean (Dyproj, Dzassoc)^6^. The DTI-ALPS indexes in both hemispheres were calculated.

### Quantification of PVS and grading of white matter lesions

PVS burden of the BG was analyzed as described previously^25^. In brief, the PVS quantification was performed in the slice containing the maximum amount of PVS in the BG^53^, of which the anterior border zones was the anterior end of the insula and the posterior border zone was the posterior end of the thalamus^24^. The PVS counting, segmentation and volume calculation were performed using the ITK-SNAP software (version 3.8, http://www.itksnap.org/). PVS number was assessed and PVS volume was calculated in both hemispheres. Following the manual delineating along the boundary of all identified PVS, the ITK-SNAP software automatically gave the voxel number of identified PVS, and PVS volume was calculated as the sum of the individual volume of identified PVS.

The WML were assessed according to the Fazekas rating scale^46^ in both periventricular white matter (PVWM) and deep white matter (DWM) on T2-weight MRI images. For each region of white matter, the ratings of participants were scored from 0 to 3. Furthermore, the total Fazekas score ranging from 0 to 6 was calculated by summing the two ratings.

### Statistical analysis

Statistical analyses were conducted with GraphPad Prism (version 9, GraphPad Inc., San Diego, CA, USA). The Shapiro–Wilk normality test was used to test the normality of variable distribution. Two sample *t*-test was applied for the comparison of continuous variables with normal distribution, Mann–Whitney test was used to compare continuous variables with non-normal distribution. Chi-squared test was used for the comparison of categorical variables. Pearson correlation analysis for normally-distributed data, whereas Spearman correlation analysis for non-normally-distributed data were performed to further evaluate the correlations between DTI-ALPS indexes and clinical characteristics, including disease duration, UPDRS, H&Y stage, LEDD^37^, MMSE, HAM-A, and HAM-D scores. To investigate whether the alteration of the DTI-ALPS index was consistent with PVS burden in PD, the relationship between the two metrics were analyzed. Pearson correlation coefficient (pcc) and Spearman correlation coefficient (scc) were calculated, respectively. *P* value <0.05 was considered statistically significant. We further performed a false discovery rate (FDR) approach for multiple testing, and FDR-adjusted *p* value < 0.05 indicated survival from multiple testing. ROC curve was used for the sensitivity analysis of DTI-ALPS indexes for PD patients. We also combined multiple biomarkers, including the DTI-ALPS indexes and PVS number/volume together into a single composite score, using multiple logistic regression^54^. Then we used the ROC curves to evaluate the quality of the composite score.

### Reporting summary

Further information on research design is available in the Nature Research Reporting Summary linked to this article.

## Supplementary information

supplementary material

Reporting Summary

## Data Availability

Clinical and neuroimaging data can be shared on reasonable request from qualified investigators by contacting the corresponding authors to the extent permitted by the Research Ethics Committee.

## Code availability

As previously mentioned in the “Methods” section, the DTI data were processed using FSL 6.0.1 (FMRIB Software Library; http://www.fmrib.ox.ac.uk/fsl). Deterministic tractography was conducted using Diffusion Toolkit version 0.6.4 and TrackVis version 0.6.1 (http://trackvis.org). The PVS quantification was performed using the ITK-SNAP version 3.8 (http://www.itksnap.org/). Statistical analyses and plots were conducted with GraphPad Prism (version 9, GraphPad Inc., San Diego, CA, USA).
