# Supplementary material for: Diffusion along perivascular spaces as marker for impairment of glymphatic system in Parkinson’s disease
Source: NPJ Parkinsons Dis. 2022 Dec 21;8:174. doi: 10.1038/s41531-022-00437-1 (PMC9772196; doi:10.1038/s41531-022-00437-1)
Supplement: Supplementary file 1 — supplementary material [file 41531_2022_437_MOESM1_ESM.pdf]

Diffusion along perivascular spaces as marker for impairment of glymphatic system in Parkinson's disease

Supplementary materials

Supplementary Table 1 Biomarkers of glymphatic system in the subgroups of patients with different onset sides.

	**Right-onset PD (n=40)**	**Left-onset PD (n=47)**	**p value**
**DTI-ALPS$_r$**	1.39 ± 0.22	1.40 ± 0.23	0.80
**DTI-ALPS$_l$**	1.38 ± 0.28	1.38 ± 0.28	0.98
**PVS BG$_r$ number**	13.80 ± 5.76	16.19 ± 9.14	0.28
**PVS BG$_l$ number**	13.36 ± 5.91	13.38 ± 9.26	0.40
**PVS BG$_r$ volume**	54.77 ± 29.28	67.09 ± 47.09	0.43
**PVS BG$_l$ volume**	50.59 ± 29.40	60.35 ± 48.22	0.57

Results are expressed as means ± SD for the continuous variables.

PD, Parkinson's disease; DTI-ALPS$_r$, right-hemispheric diffusion tensor image analysis along the perivascular space; DTI-ALPS$_l$, left-hemispheric DTI-ALPS; PVS, perivascular space; BG$_r$, right-hemispheric basal ganglia; BG$_l$, left-hemispheric BG.
